# Active Transport of Therapeutic Triblock Amphiphilic Polymer Poloxamer 188 in Brain Endothelial Cells for Cellular Repair

**DOI:** 10.4236/jbise.2025.187022

**Published:** 2025-07-30

**Authors:** Nagham Alatrash, Anne Alsup, Mia Grubbs, Vanessa Nomellini, Michael Cho

**Affiliations:** 1 Department of Bioengineering, University of Texas at Arlington, Arlington, TX, USA; 2 Department of Surgery, University of Texas Southwestern Medical Center, Dallas, TX, USA

**Keywords:** Poloxamer 188, Transport Mechanisms, Computer Vision Pipeline, Blood-Brain Barrier, Cellular Repair Machinery

## Abstract

Amphiphilic triblock poloxamer 188 (P188) has demonstrated its therapeutic potential for muscle, cardiac and neurological injuries. While this surfactant is thought to primarily reseal the disrupted cell membrane, the specific mechanisms that mediate the reparative effect of P188 remain to be fully elucidated. Here, we investigated the transport mechanisms of P188 cellular uptake by fluorescently conjugating P188 with the fluorophore, Rhodamine 110 (Rh110). Fluorescent conjugation did not alter the P188 structure as characterized by nuclear magnetic resonance, Fourier Transform infrared spectroscopy, and acid-base titration, and the hydrophobicity was also quantified. In mouse brain endothelial cells, Rh110 alone was unable to accumulate inside the cells, while the P188 + Rh110 was rapidly transported across the cell membrane and became saturated in less than 1 hour. The transport dynamics were determined to be clathrin-dependent endocytosis, which was significantly altered in saponin-damaged cells or in cells with disrupted actin cytoskeletal organization; this suggests that transport via vesicle trafficking may be involved. Reparative effects of P188 appear to remodel the membrane organization and restore the transport properties. Instead of relying on manual image analysis, we utilized a machine learning pipeline that was recently developed in our laboratory to more rapidly and accurately analyze the cellular images of fluorescent P188 dynamics. This computer vision pipeline significantly reduced the time needed to segment, analyze, and perform statistical analyses. Finally, when injected into the mouse tail vein following a traumatic injury to the brain, we report for the first time that the P188 + Rh110 was observed in the brain tissue, indicating that P188 can cross the blood-brain barrier (BBB). Taken together, the dual therapeutic effects of P188 should include (1) resealing the disrupted cell membrane and (2) modulation of the intracellular cell repair machinery that might be involved in response to traumatic brain injury.

## 1. INTRODUCTION

Brain endothelial cells (BECs) are known to regulate the blood-brain barrier (BBB), which plays a key role in controlling the transcellular and paracellular transport due to tight junctions between adjacent cells [1]. Brain injuries induced by trauma may lead to neurological dysfunction that is associated with an altered BBB. We have previously demonstrated that a shockwave blast can structurally and functionally disrupt the brain endothelium, suggesting the altered BBB likely gives rise to traumatic brain injury (TBI). Considering the central role of BECs as the primary anatomical and physiological component of the BBB, an *in vitro* endothelium model is used in this study to elucidate the effects of altered BBB structure and function. Unlike neurons or glial cells, BECs form the selective barrier that tightly regulates the passage of ions, molecules, and immune cells between the bloodstream and brain tissue, largely through specialized tight junctions and unique transport mechanisms [2, 3]. In TBI, disruption of the BBB mediated by dysfunctional BECs leads to an increased permeability. This allows neurotoxic substances and inflammatory cells to infiltrate the brain parenchyma and exacerbate secondary injury processes [4, 5]. While neurons and glia are critical for brain function and are involved in TBI pathology, their roles are downstream or indirect in BBB maintenance. BECs, by contrast, directly determine barrier integrity and are uniquely positioned to mediate both injury-induced dysfunction and potential repair mechanisms [4–6]. Therefore, studying BECs can provide direct insight into BBB breakdown and its contribution to TBI pathology, offering opportunities for the development of targeted biomarkers and therapeutic strategies that might be overlooked by focusing solely on neurons or glial cells.

Currently, there are no treatment or preventive measures that specifically target TBI due to the selectivity of the BBB [1]. However, we recently showed that FDA-approved amphiphilic triblock copolymer poloxamer 188 (P188) is capable of repairing cellular damage in response to traumatic injury [1]. Delivered either in a free solution or encapsulated in nanoparticles, P188 treated a lesion that was created in an *in vitro* endothelium model by shockwave-induced TBI. It was further supported by a down-regulation of the tight junction markers was restored, the permeability through the endothelium returned to normal, and the expressions of the matrix-degrading matrix metalloproteinases (e.g., MMP2 and MMP9) were suppressed. Specific mechanisms by which P188 facilitates cell repair remain to be fully elucidated [2].

Surfactants, including P188, generally reduce surface tension. There are several well-known surfactant-like molecules that are found inside cells, such as phospholipids, cholesterol, antimicrobial peptides, and lipid-anchored proteins [7, 8]. While the potential therapeutic effects of P188 have been thought to be limited to resealing the damaged cell membrane, P188 could be internalized and contribute to the repair of the injured cells, similar to the reparative role of intracellular surfactant-like molecules and proteins. In addition to stabilizing the membrane, intracellular surfactants respond to stress, injury, or disease [9–12]. Therefore, to further determine the potential role of P188 leading to cell rescue and repair, the transport mechanisms that mediate P188 uptake should be determined. In this work, we have conjugated P188 with the zwitterionic Rhodamine 110 fluorophore (Rh110) to monitor the cellular uptake of P188 copolymer over the course of time and determine the cellular uptake mechanisms. Our results indicate that the fluorescently conjugated P188 (referred to as P188 + Rh110) utilizes the well-known clathrin-dependent endocytosis to enter the cell, and such transport requires an intact actin cytoskeleton network.

Because the transport dynamics of P188 + Rh110 were typically tracked in real-time through live cell imaging, a large number of cellular images were acquired. In the past, laborious manual analysis of images was performed to identify the cell boundaries by hand, and thus severely limited the number of cells that could be analyzed rapidly and accurately. In addition, manual segmentation frequently requires multiple researchers to minimize any potential bias and thus further poses difficulties with image analyses. To overcome this challenge, we developed a computer vision pipeline [13] that automated the cell segmentation to perform faster and statistically more accurate data analysis. The pipeline has led us to probe and validate/refute hypotheses that otherwise would have been difficult to formulate based on laborious and manual analyses. This machine learning pipeline has been successfully applied to analyze the calcium dynamics in pancreatic cells. In one study, the pipeline determined that manual image analysis was underestimated [13] while in another study, the manually analyzed results were overestimated [14]. Such inconsistency was noticeable, and therefore, the machine learning pipeline was utilized in the current study.

## 2. MATERIALS AND METHODS

### Reagents

Poloxamer 188 was purchased from Maroon Biotech (Chicago, IL) and Sigma Aldrich (St. Louis, MO). 4-Dimethylaminopyridine, 1,4-Dioxane, Succinic anhydrous, triethylamine, N,N-Diisopropylethylamine, NaOH, pyridine, sodium azide (NaN_3_), 2-deoxy-D-glucose (2DG), sucrose, Dynasore hydrate, nystatin, and phosphate buffered saline 10X were purchased from Sigma Aldrich. All the cell medium and additives were purchased from Cell Biologics Inc. (Chicago, USA). The alamarBlue^®^ cell viability method (Bio-Rad, Life Science, Hercules, CA, USA) was used to determine cell proliferation. MemBrite^®^ Fix Cell Surface Staining Kits were purchased from Biotium, Inc. (Fremont, CA).

### Synthesis Of P188-COOH

The COOH-terminated P188 was synthesized using the modified previous procedure. Briefly, 4.50 g of P188, 0.27 g of Succinic anhydrous, 0.14 g of 4-Dimethylaminopyridine, and 0.25 mL of triethylamine were dissolved in 15 mL of 1,4-Dioxane and stirred at room temperature for 24 hours. Subsequently, the mixture was subjected to rotary evaporation until a milky color was formed, after which ether was added to induce precipitation. The resulting product was then isolated through filtration.

### Synthesis of P188 + Rh110

Synthesized P188-COOH (200 mg) and Rh110 (19 mg) were dissolved in 10 mL of pyridine and stirred at room temperature for two days. Then, 26 equivalent N,N-Diisopropylethylamine were added, and the mixture was stirred for 6 hours. The resulting product was purified using a dialysis tube with a molecular weight cutoff of 3.5K against 20% ethanol for five days, followed by lyophilization.

### Cell lines and culture conditions

C57BL/6 Mouse Primary Brain Microvascular Endothelial (MBEC) cells were purchased from Cell Biologics Inc. MBEC cells were grown and maintained in a complete mouse endothelial cell medium supplemented with Kit (catalog # M1168). The Cells were incubated at 37°C within a humidified atmosphere of 5% CO_2_.

### Cell Viability Assay

Cell growth and viability were evaluated by the alamarBlue assay. MBEC cells were seeded in 96-well plates at a density of 1 × 10^4^ cells per well and incubated for 24 hours. Subsequently, P188 was introduced into the wells at the concentrations of 10 μM, 100 μM, and 500 μM. After an additional 24 hours of incubation, fluorescence measurements were obtained using Synergy^MT^ HT multi-mode microplate reader (Bio-TeK, Vermont, USA), with excitation and emission wavelengths set at 530 nm and 590 nm. The alamarBlue was prepared at a 1:10 dilution following the manufacturer’s protocol.

### Cellular Uptake

MBECs Cells were cultured in standard conditions and were treated with 10 mM of P188 + Rh110 for 30 min at 37°C or 4°C. Fluorescence images were acquired at 1 min intervals using an inverted fluorescence microscope (Nikon Eclipse Ti).

### Active Transport Channels

MBEC Cells were cultured under standard conditions in the presence or absence of 10 mM sodium azide (NaN_3_) and 2-deoxyglucose (2DG) (active transport energy inhibition), 1 M sucrose (clathrin-mediated endocytosis inhibitor), 50 μM Nystatin (a lipid raft-mediated endocytosis inhibitor), and 300 μM Dynasore (GTP-protein active receptor-mediated endocytosis inhibitor) for a duration of 30 min prior to treatment with P188 + Rh110. Nuclei were counterstained with DAPI. In the calcium channel blocker experiments, cells were cultured under standard conditions with 10 μM of Verapamil or Gadolinium for 30 min each. All images were obtained by an inverted fluorescence microscope (Nikon Eclipse Ti).

### Tail vein injection of P188 + Rh110

Male and female CD1 mice at 11-12 weeks old were anesthetized with 3% isoflurane (using a nosecone device) and restrained, then the hair was removed from their head, and a surgical incision was made in their scalp to expose the skull. A heating pad was placed underneath the animal to regulate its body temperature during the TBI procedure. A single cortical impact was then delivered utilizing a Benchmark Stereotaxic Impactor (Leica Microsystems, Wetzler, Germany) to create a moderate TBI using the pneumatic cylinder at a velocity of 4.5 m/sec. Buprenorphine (0.05 mg/kg) was given intraperitoneally immediately following injury for analgesia, and the incision on the head was closed. Sham-injured mice were undergoing all the above procedures, except for the cortical impact. After 1 hour, each mouse received an intravenous injection via the tail of a vehicle or 15 μg of P188+Rh110. Animals were randomly divided into four groups: (1) sham + vehicle, (2) sham + P188, (3) TBI + vehicle, and (4) TBI + P188. Mice were euthanized after 24 hours, and brains were harvested and fixed in 10% neutralized buffer formalin.

### Immunohistochemistry

Following the fixation steps, paraffin sections were cut at 20 μm thickness and fixed to the microscope slides. Subsequently, brain sections underwent staining with Nissl stain (green) for the identification of neurons and DAPI (blue) for the visualization of nuclei. The stained slides were acquired with an inverted fluorescence microscope (Nikon Eclipse Ti). All images were acquired using a 20X microscope objective.

### Statistical analysis

All experiments were replicated at least three times. Data are presented as mean ± SEM, and p < 0.05 was indicated with an asterisk (*), whereas p < 0.001 was indicated with three asterisks (***). The machine learning pipeline automatically performed an ANOVA as well as a Dunnett’s test with experiments that consisted of more than 2 images, assuming one image was the control sample, and all others were experimental. ANOVA, Dunnett’s test, and two-sample t-test were performed before normalization.

## 3. RESULTS

### Characterization of fluorescently conjugated P188

**Figure 1.**
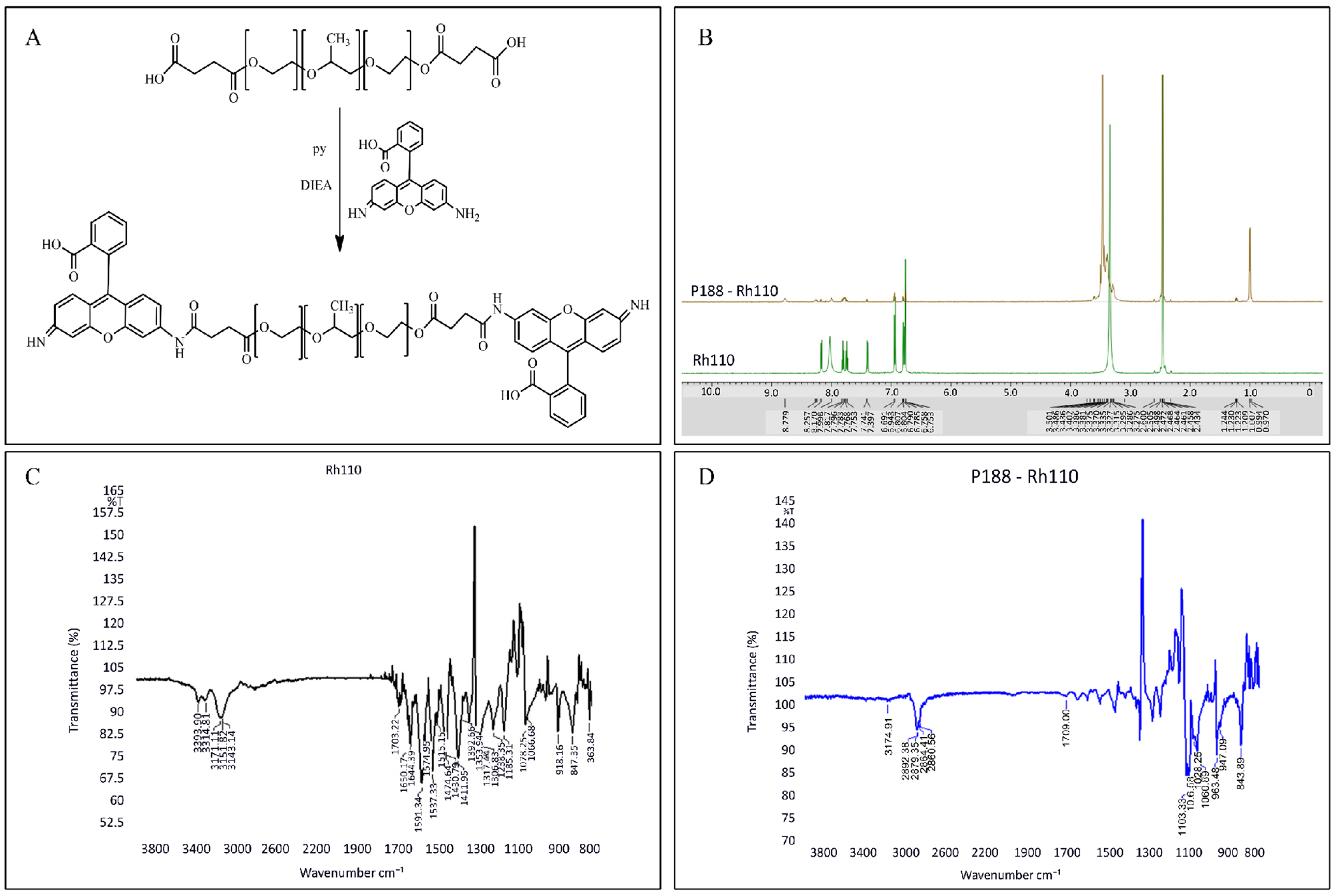
Synthetic scheme of P188 + Rh110 conjugate by applying N,N-Diisopropylethylamine (DIPEA, Hunig’s base) coupling chemistry (A). ^1^H NMR in DMSO-d6 of P188 and conjugated P188 + Rh110 were obtained on a JEOL Eclipse 500 MHZ spectrometer (B). FTIR spectra of Rh110 and conjugated P188 + Rh110 (C).

Analytical techniques and methodologies were utilized to determine the synthesis and characterization of FDA-approved P188. First, N,N-Diisopropylethylamine (DIPEA, Hunig’s base) coupling chemistry [15] was applied to conjugate the Rh110 fluorophore to P188 (Figure 1(A)). Briefly, the hydroxyl terminal groups of P188 were converted to carboxylic groups (-COOH). Then, activated carboxylic acid groups were directly conjugated to primary amines (-NH_2_) of Rh110 fluorophore via amide bonds (Figure 1(A)). Following the synthesis of P188 + Rh110, the conjugated P188 was characterized using several techniques. First, the chemical structure of Rh110 alone or conjugated P188 + Rh110 was confirmed by nuclear magnetic resonance (NMR) spectroscopy. ^1^H NMR was obtained on a JEOL Eclipse 500 MHZ spectrometer (Figure 1(B)). Second, Rh110 and P188 + Rh110 copolymers were characterized using a Fourier Transform Infrared spectrophotometer (FTIR) (IRPrestige-21, SHIMADZU Institute, University of Texas at Arlington). Spectroscopy measurement was carried out to probe changes in the chemical structure of P188 + Rh110 (Figure 1(C)). FTIR spectra demonstrated that the P188 + Rh110 had the same carbonyl stretching vibration at about 1709 cm^−1^ as the Rh110 shows. In addition, the P188 + Rh110 was characterized by a new absorption peak at 3124 cm^−1^ and 1680 cm^−1^. Third, to confirm that the Rh110 was conjugated on both sides of P188, we conducted a titration experiment using NaOH as a base and phenolphthalein as an indicator in an acid-base titration experiment. This acid-base titration is a method of quantitative analysis for determining the concentration of the carboxylic acid in the P188 + Rh110 by neutralizing it with a standard solution of a known concentration, NaOH. Based on the calculation, we can confirm that there are two COOH terminal groups on the P188 + Rh110. Finally, we determined the partition coefficient to examine the solubility of P188 + Rh110 by measuring log *P* in octanol/PBS buffer mixtures (*P*_o/b_). The value of log *P_o/b_* was found to be ~ −0.14, indicating that the conjugated P188 + Rh110 partitioned between lipid and aqueous phases with a little more affinity to the aqueous phase. Taken collectively from the characterization studies, the structure and function of P188 + Rh110 are unlikely to be altered by conjugation of the fluorophore.

### Cell Viability and Cellular Uptake

We next performed cell viability experiments using mouse brain endothelial cells (MBECs). Cells were treated with 10, 100, or 500 μM P188 for 24 hours, and the viability was determined using the alamarBlue assay. In the range of P188 concentrations we applied to determine cell viability, no statistical difference was observed, indicating that P188 does not adversely affect cell viability. We also confirmed that the fluorescent conjugation did not change the cell viability (data not shown). Based on these findings, we chose to use the minimum 10 μM P188 concentration throughout the manuscript, unless indicated otherwise.

**Figure 2.**
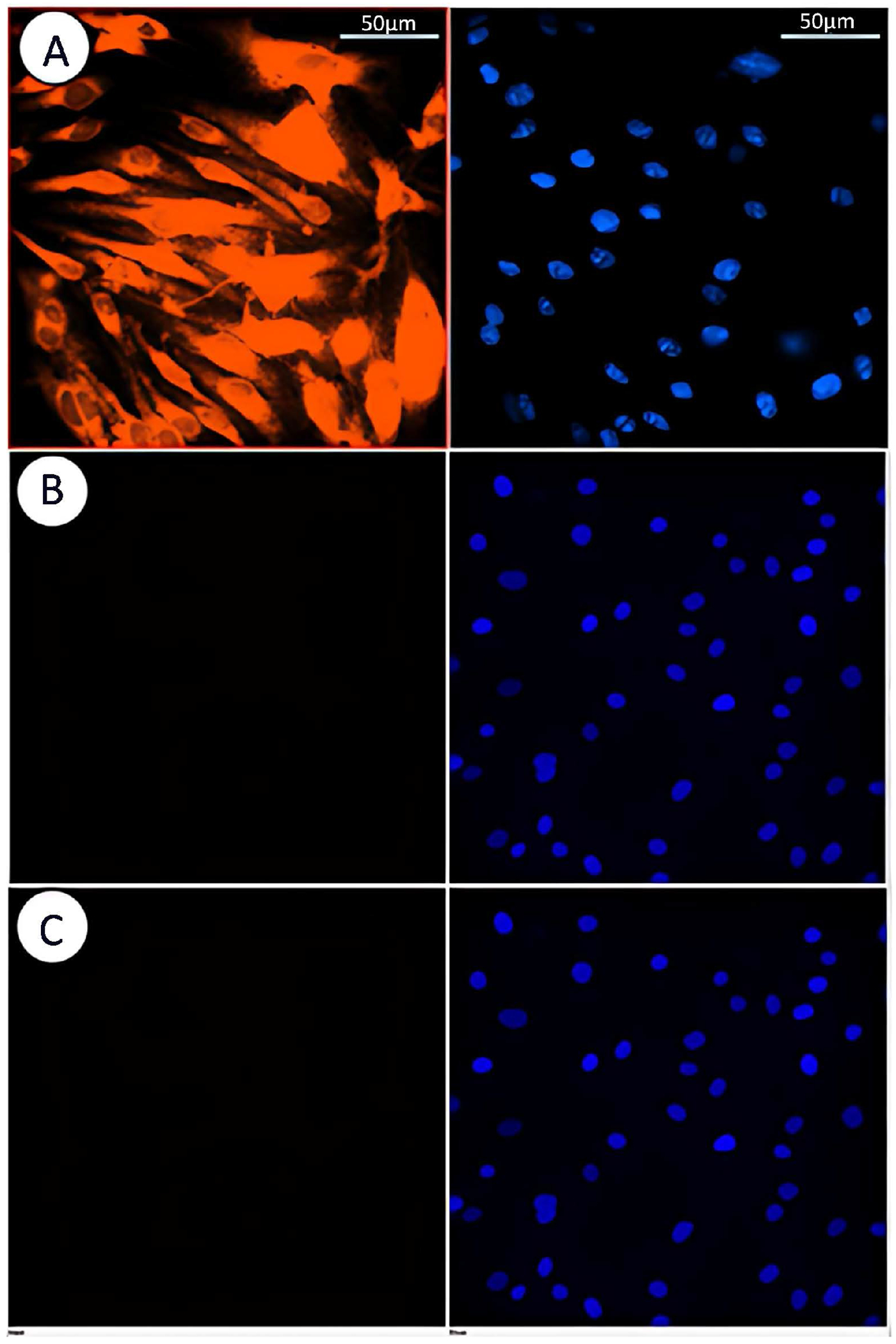
MBECs were treated for 30 min with 10 μM of P188 + Rh110 (A), with 10 μM Rh110 alone (B), and treated with a mixture of 10 μM P188 and 10 μM Rh110 (C). No detectable fluorescence signals were acquired in (B) or (C). Each companion image of DAPI-stained nuclei is shown to the right. Scale bar = 50 μm.

Live cell microscopy was performed to monitor the accumulation of P188 + Rh110 (10 μM) inside MBECs (Figure 2(A)). In contrast, there was no accumulation of the zwitterionic Rh110 alone (10 μM) inside the cells (Figure 2(B)), indicating Rh110 was unable to cross the cell membrane or rapidly effluxed [16]. Moreover, when cells were treated with a mixture of unconjugated P188 and Rh110 (10 μM each), the results also showed no cellular uptake (Figure 2(C)), although we cannot completely rule out cellular uptake of unlabeled and unconjugated P188.

### Time Lapse Experiment

**Figure 3.**
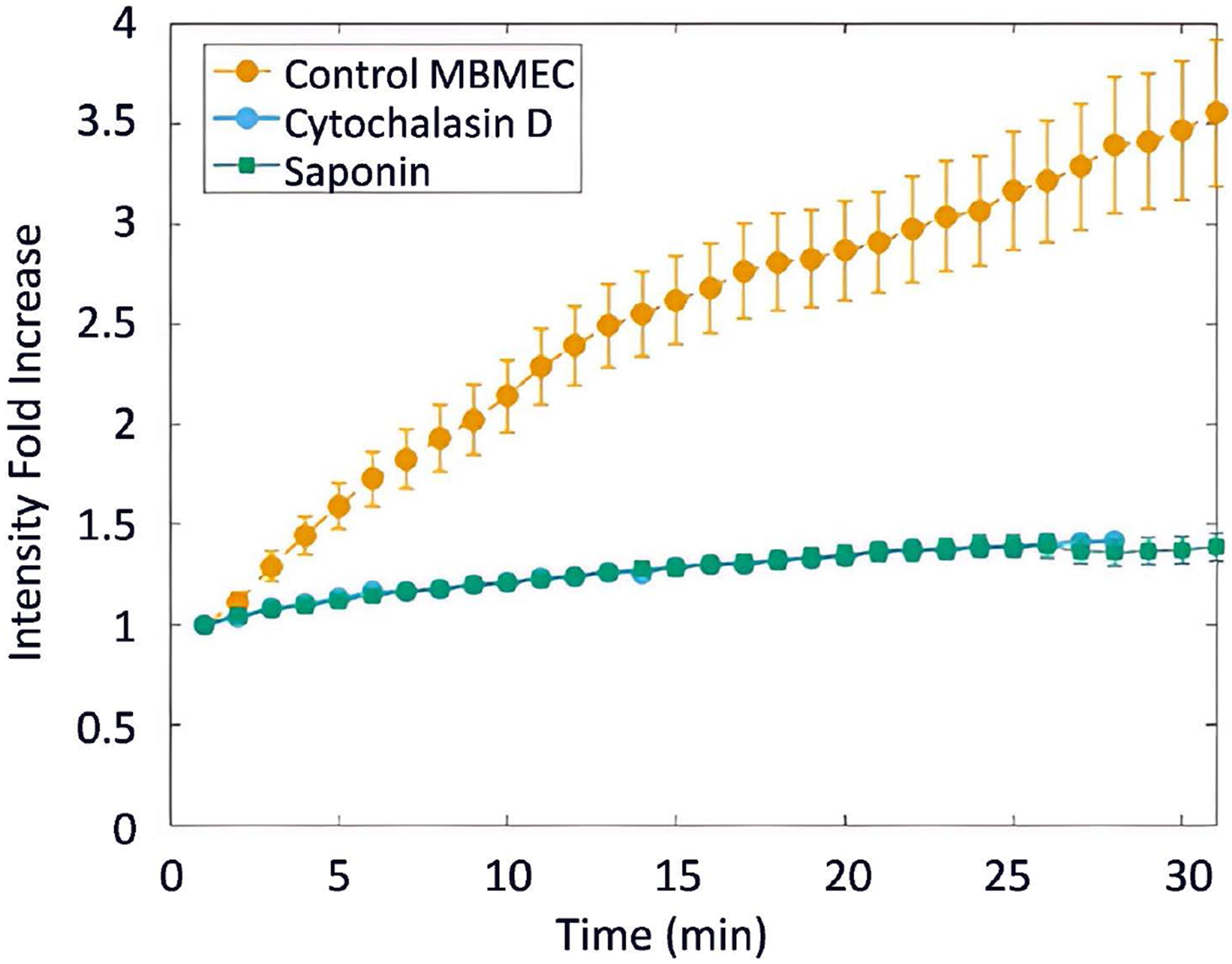
Analysis of time-dependent accumulation of P188 + Rh110 by applying the machine learning pipeline. The automated cell segmentation and subsequent statistical analysis required <15 min for each experiment. To maintain consistency across all time-lapse experiments at 1 min intervals, regions of interest (ROIs) from the first frame are averaged to obtain a baseline value. The intensity values from all frames are then normalized by this baseline. These normalized values are subsequently averaged for each frame to track changes over time. Changes in cell size are inherently accounted for, as mean intensity is calculated by dividing the total pixel intensity by the number of pixels within each cell. Background subtraction was not applied due to the heterogeneous distribution and movement of P188 + Rh110 in the solution. The accumulation was rapid in control cells and continued to grow to an approximately 4-fold increase in a 30-min observation time. The cell membrane permeabilization or actin disorganization appears to inhibit the transport of P188 + Rh110. Data represent mean ± SEM (n = 3 independent experiments under each of the three conditions) of 40 to 52 cells. The number of cells in single experiment ranged from 12 to 18.

Analysis of time-lapse experiments can be performed to determine the dynamics of P188 transport in a short (30 min) or longer time (24 hours). Rapid and accurate analysis of hundreds of individual cells is made feasible by the implementation of the recently developed machine learning pipeline [13]. We acquired the images of P188 for 30 min at 1 min intervals and analyzed the accumulation of P188 in MBECs under 3 different experimental conditions, *i.e.*, control cells and cells treated with either Cytochalasin D or saponin (Figure 3). In control cells, the P188 rapidly accumulated inside the cells and reached ~4-fold increase in the fluorescent intensity and became saturated. In contrast, the cells treated with either Saponin or Cytochalasin D prevented the transport of P188 + Rh110. These findings support the postulate that the P188 transport via endocytosis is disrupted by either cell membrane permeabilization or actin cytoskeletal reorganization.

**Figure 4.**
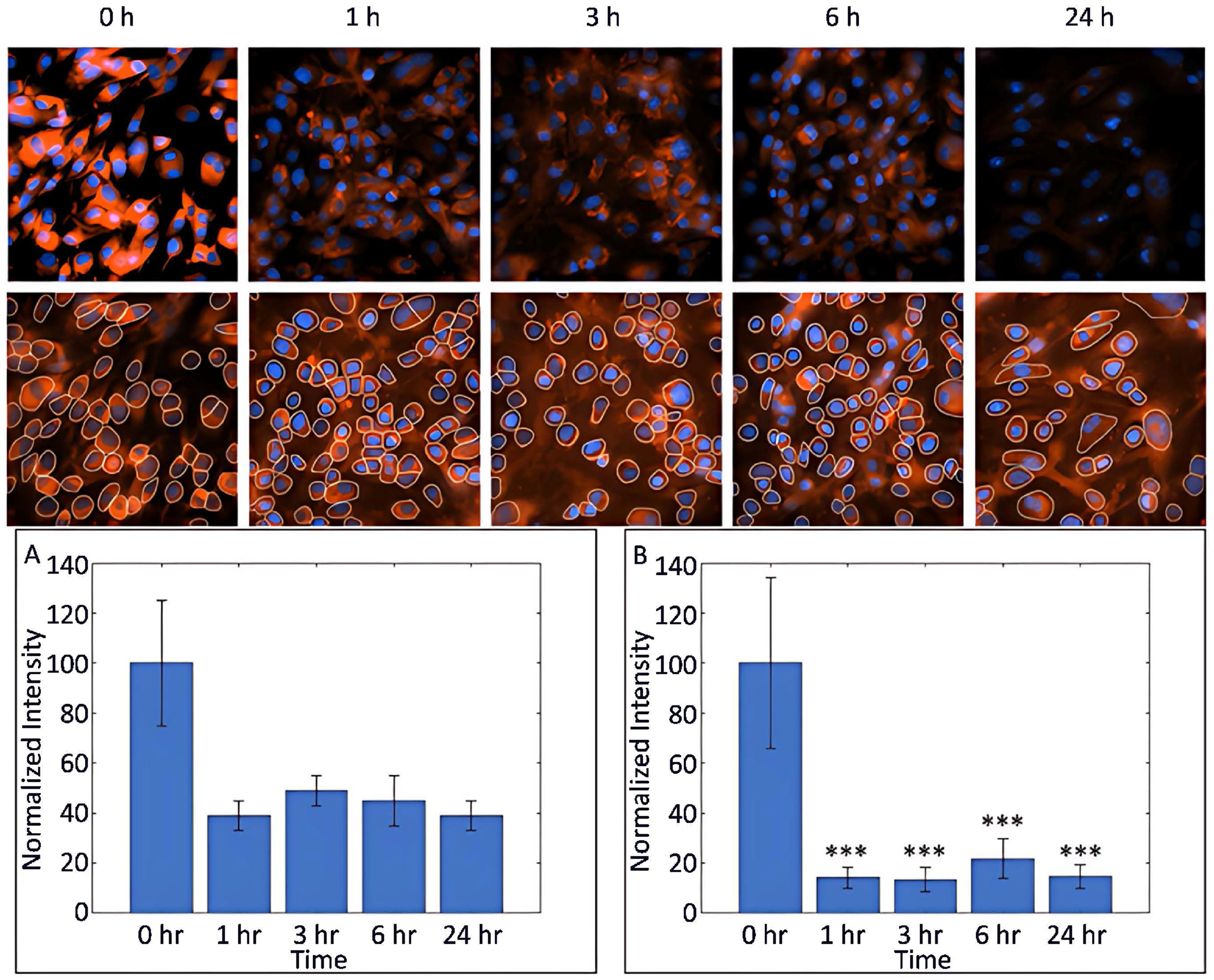
Time-lapse images of P188 + Rh110 in MBECs at various time points. Cells were treated with P188 + Rh110 and incubated at 37°C for the indicated time. Unprocessed images of P188 + Rh110 in MBECs (top row) and individual cells in the same images were segmented by the computer vision algorithm (bottom row). Nuclei were counter-stained with DAPI. Quantitative analysis of the cellular uptake/exocytosis of P188 + Rh110 by (A) manual cell segmentation or (B) using the automated computer vision pipeline. Data represent the mean ± SEM (n = 3 independent experiments). ***p < 0.001.

We extended the transport dynamics studies by monitoring the P188 concentration in the cells over 24 hours. As shown in Figure 4, a lower magnification microscope objective was used to capture multiple cells. The images shown on the top row illustrate un-segmented cells at the time points of 0, 1, 3, 6, and 24 hours. The unprocessed raw images provide a qualitative analysis that the early phase 4-fold increase in the P188 concentration inside the cells (see Figure 3) is significantly diminished in just 1 hour. The bottom row of images represented the individual cells that were segmented by the computer vision pipeline. Moreover, we quantitatively determined the exocytosis of P188 and compared the results that were analyzed manually or by applying the computer vision algorithm. The findings are consistent in that, after entering the cells, P188 is also effluxed and/or exocytosed within 1 hour, and ~20% of the P188 + Rh110 conjugates remain in the cells over a 24-hour observation period.

### Active Transport Mechanisms

**Figure 5.**
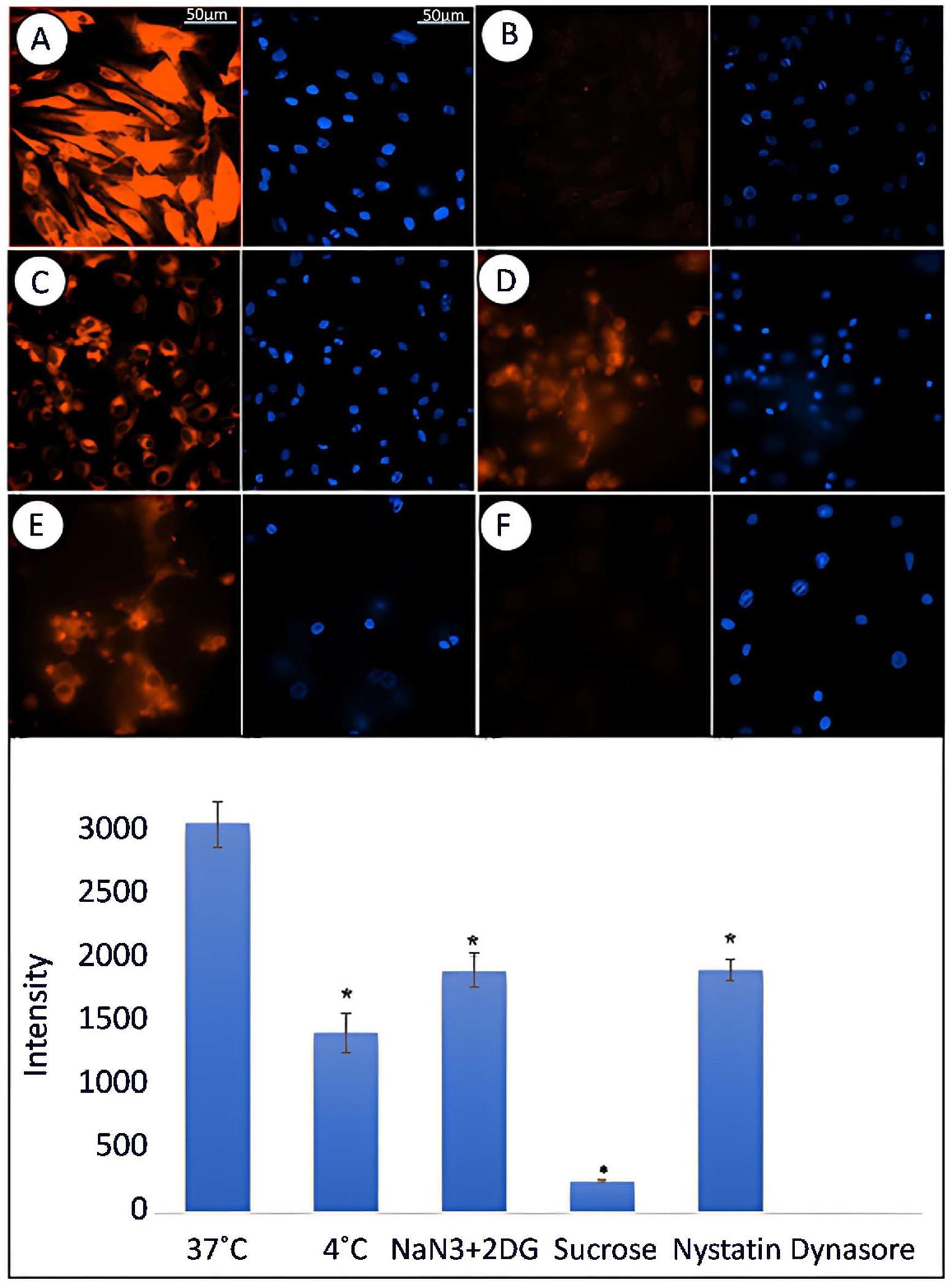
MBECs were treated with 10 μM of P188 + Rh110 for 30 min at 37°C (A) or at 4°C (B). Cells were incubated with 10 mM Sodium Azide (NaN_3_) and 2-deoxy-D-Glucose (2DG) (active transport energy inhibition) (C), 1 M Sucrose (clathrin-mediated endocytosis inhibitor) (D), 50 μM Nystatin (a lipid raft-mediated endocytosis inhibitor) (E), or 300 μM Dynasore (GTP-protein active receptor-mediated endocytosis inhibitor) (F) for 30 min prior to treatment with P188 + Rh110. Nuclei were counterstained with DAPI. All images were obtained after 30 min of treatment with P188 + Rh110. Scale bar = 50 μm. Quantitative analysis was performed under each experimental condition. Data represent mean ± SEM (n = 3 independent experiments). *p < 0.05.

There are several mechanisms by which molecules can enter cells, such as facilitated diffusion, passive diffusion, active transport, and endocytosis [17]. Structural characteristics of the molecule (e.g., net electrical charge and size) and microenvironment (e.g., temperature) play an important role in the cellular uptake and subcellular localization [18]. As demonstrated above, the P188 + Rh110 was rapidly accumulated inside the MBECs and became saturated in <60 min at 37°C (Figure 5(A)). The transport of conjugated P188 + Rh110 was prevented when the cells were incubated at 4°C (Figure 5(B)), suggesting the transport of P188 across the cell is not simply through passive diffusion, but instead active transport and endocytosis may be involved [19–21]. In the presence of active transport inhibitors, however, the cellular uptake of P188 + Rh110 was partially or completely inhibited. For example, sodium azide (NaN_3_) and 2-deoxy-D-glucose (DOG), which are known to inhibit energy-dependent active transport [22, 23], were used to show that the cellular uptake of P188+Rh110 was partially inhibited (Figure 5(C)). Endocytosis is known to be mediated by at least 3 different pathways. First, a clathrin-mediated endocytosis was inhibited by Sucrose [24] and shown to reduce the P188 accumulation by >80% (Figure 5(D)). Second, another endocytosis pathway is the lipid raft-mediated endocytosis. Using the inhibitor Nystatin for this pathway [25], the P188 accumulation was decreased by ~30% (Figure 5(E)). Third, a nearly full inhibition of P188 + Rh110 accumulation was observed in cells treated with Dynasore (Figure 5(F)), a GTP-protein active receptor-mediated endocytosis inhibitor [24, 26]. The fluorescence intensity in response to the Dynasore treatment was below the detectable level. Quantitative fluorescent intensity measurements are summarized in a bar graph. Collectively, the inhibition of endocytic pathways indicates that the P188 uptake was likely transported via endocytosis and more dominantly by the dynamin-dependent endocytic trafficking. We also examined the potential role of ion channels that might be involved in the P188 transport. Treatment with 10 mM Verapamil to block the voltage-operated calcium channels did not alter the P188 cellular uptake. However, treating the cells with 10 μM Gadolinium (a known mechanochannel blocker) induced a partial inhibition of the uptake of P188 + Rh110 (data not shown).

### Cellular uptake of P188 in saponin-damaged MBECs

**Figure 6.**
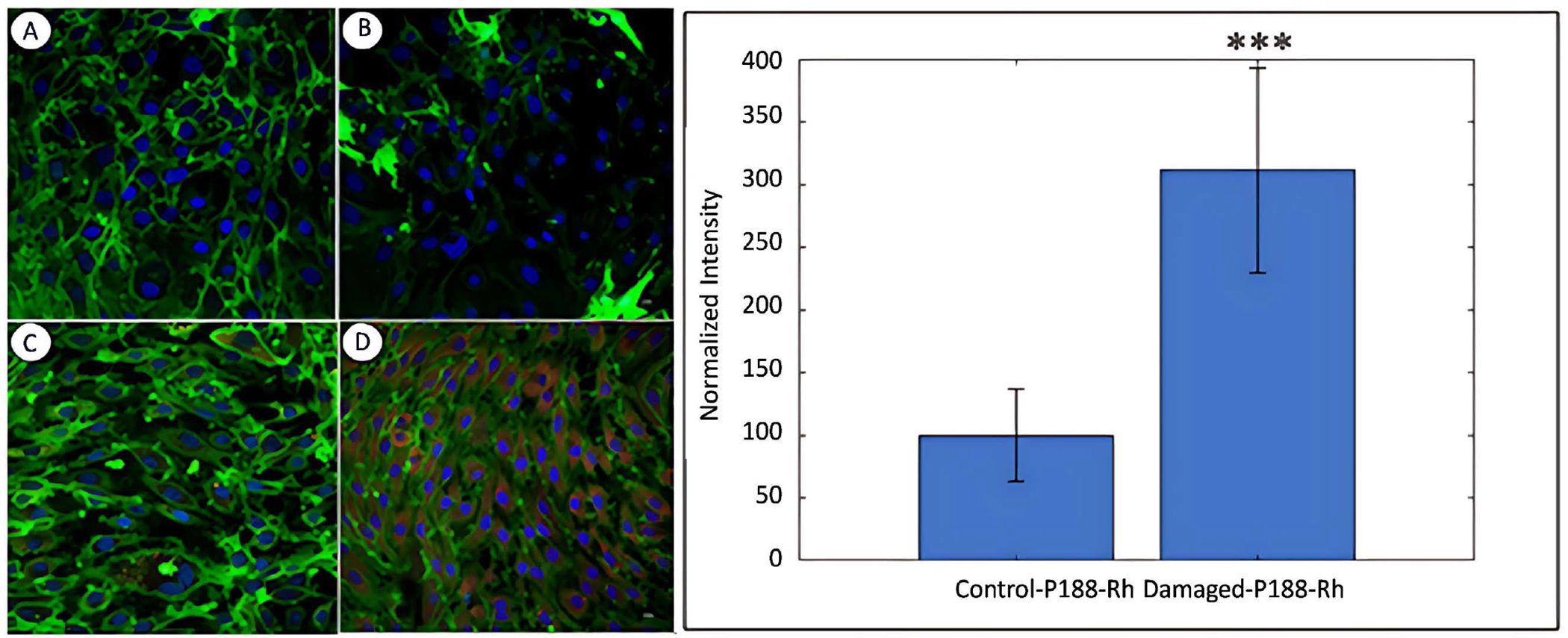
The cell membranes were visualized using Membrite (green) and nuclei identified by DAPI (blue). The control cells show well-defined cell boundaries in a monolayer (A). In response to a Saponin 0.01% treatment for 5 min, the cell membranes were essentially non-recognizable, although the nuclei were visible (B). The P188 did not alter the cell membranes in normal healthy MBECs (C). When damaged by saponin and then treated with P188, the cells show a remarkable recovery of their cell membrane (D). Scale bar = 50 mm. Quantitative analysis using the computer vision algorithm shows P188 was retained in the repaired by 3-fold (bar graph). Data represent the mean ± SEM (n = 3 independent experiments). ***p < 0.001.

The next step was to determine whether the repair of damaged cells could be achieved through the restoration of the cell membrane following an injury. Using a cell membrane-specific dye (Membrite), MBECs were damaged by incubating them with saponin, and their cell membrane was fluorescently imaged. As expected, control cells demonstrate well-defined cell boundaries (Figure 6(A)). In fact, this image also illustrates a monolayer formed by the MBECs. However, when treated with 0.01% Saponin for 5 min, the cell membranes become almost unrecognizable (Figure 6(B)). In order to test the effect of P188 on normal healthy cells, control MBECs were incubated with P188 + Rh110, and then the cell membranes were visualized (Figure 6(C)). P188 + Rh110 does not alter the membrane of normal cells, and interestingly, P188 that entered the normal healthy cells was barely visible (see Figure 6(C)). Finally, when the Saponin-damaged cells were treated with P188 + Rh110, not only the cell membranes were restored to that observed in control cells, but the repaired cells also retained a significant amount of P188 + Rh110 even after 48 hours following the P188 + Rh110 treatment (Figure 6(D)). Again, the computer vision algorithm was applied to rapidly analyze the images and determined that there was a 3-fold increase in the retention of P188 + Rh110 in the repaired cells.

## 4. DISCUSSION

P188 has long been recognized as an FDA-approved amphiphilic triblock copolymer that can reseal the damaged cell membrane [12]. Potential clinical benefits of P188 have been examined in numerous studies, including central nervous disorders, Parkinson’s disease, Amyotrophic Lateral Sclerosis, and TBI [27–29]. However, questions have been posed in the past whether P188 can enter the cell and, if it does, what reparative mechanisms are modulated by P188. The first step towards answering such questions is to demonstrate that P188 is capable of transporting across the cell membrane. Therefore, this study was focused on conjugating P188 with a Rh110 fluorophore to monitor and track its transport dynamics.

Conjugated P188 + Rh110 is found to readily enter the brain endothelial cells through an active energy-dependent transport process across the cell membrane. As the energy was depleted by metabolic inhibitors or lowering temperature (4°C), the cellular uptake was diminished, suggesting the transport is not simple diffusion but rather through active biological processes such as endocytosis. Endocytosis is mediated by three different pathways: clathrin-dependent, lipid raft-mediated, and dynamin-dependent. Pharmacological inhibitors were used for each of the three known pathways. While all three pathways appear to be involved, inhibition of the dynamin-dependent pathway completely blocked the P188 transport (see Figure 5). Consistent with this observation, the P188 transport also requires intact cytoskeletal organization. One can speculate that P188 may have dual reparative effects. First, as repeatedly demonstrated in the past, it can reseal the membrane defects temporarily [9, 11, 12]. Second, the internalized P188 promotes downstream pathways, including the down-regulation of matrix-degrading enzymes [1] and inhibition of protein aggregation and denaturing [30]. Our results in this paper support the postulate of dual reparative effects.

**Figure 7.**
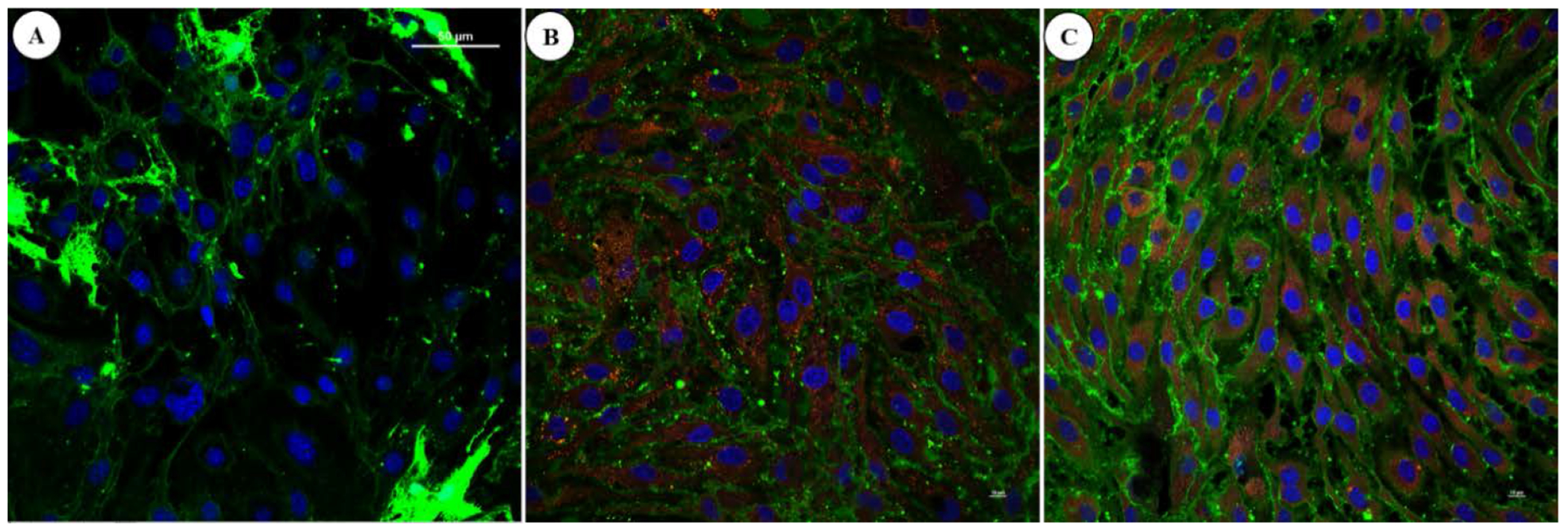
Three-color images of the cell membrane (green), P188 + Rh110 (red), and nuclei (blue). Saponin-damaged MBECs after 48 hr show a loss of the cell membrane and no retention of P188 + Rh110 (A). When treated with P188 + Rh110 (10 mM) for 18 hr, cell repair becomes visibly convincing (B), and a treatment with P18 + Rh110 for 48 hr indicates a recovery of endothelial cells and formation of a monolayer (C). Unlike the untreated cells, the repaired cells appear to have retained P188 + Rh110. Scale bar = 50 μm.

Conjugated P188 + Rh110 was cleared from the control cells in 24 h compared to the damaged cells. When the damaged cells were treated with P188 + Rh110, however, it remained inside the cells and showed evidence of cell repair after 48 h, including restoration of the cell membrane and formation of a monolayer of endothelial cells (Figure 7). At least three postulates could be formulated. First, in the damaged cells, the cell membrane is permeabilized and no longer capable of regulating the influx/efflux pathways (Figure 7(A)). The dysfunctional cell membrane offers a viable explanation of the P188 + Rh110 complex to diffuse into the cell and then diffuse out of the cell once a reverse gradient of the P188 concentration is established. Second, the efflux pump has been demonstrated in the extrusion of drugs out of the cells. It is an important area of investigation, especially in antibiotic-resistant bacteria [31]. However, the efflux transporters require energy because they must pump the substances against their concentration gradient. While this efflux pump mechanism cannot be readily ruled out, it may be unlikely to be responsible for the extrusion of P188. In the damaged cells, the ATP production is stressed [32] and insufficient to sustain such an extrusion. Third, the cells undergoing repair are shown to retain P188 and are capable of reforming a monolayer (Figure 7(B) and Figure 7(C)), suggesting both the structural and functional properties of the endothelium are restored. Although additional experiments are needed to conclusively confirm the cell repair mechanisms through the P188-dependent cell membrane resealing, we have demonstrated that this potential mechanism could be monitored in real-time.

Interestingly, conjugated P188 + Rh110 appears to co-localize with lysosomes. Lysosomes are membrane-bound organelles that are involved in various cell processes, including degradation and recycling cellular waste, cellular signaling, and energy metabolism [33–37]. Lysosomes not only serve as a cellular recycling center, but they are also involved in energy homeostasis, generating building blocks for cell growth, mitogenic signaling, priming tissues for angiogenesis and metastasis formation, and activating transcriptional programs [38]. Recent studies have shown that lysosomal damage activates cellular repair mechanisms to protect against lysosomal membrane permeabilization-induced cell death [39]. Preliminary co-localization experiment results do suggest that P188 could accumulate in lysosomes and interfere with the endosomal pathway and the intercellular membrane. While additional experiments are underway to further elucidate the involvement of lysosomes in the P188-mediated cell repair mechanisms, it is hypothesized that P188 is endocytosed and may be co-localizing with intracellular organelles such as lysosomes and mitochondria (preliminary images not shown) that are known to be involved in the cell repair machinery [40]. The lysosome pH is between 4.5 and 5. This acidic environment is necessary to enable the function of the lysosome enzymes [41–43]. We therefore tested dissociation of Rh100 from P188 by incubation the Rh110 + P188 complex in different pH solutions and found no statistically significant decrease in fluorescence intensity. For example, an incubation of the complex for 60 min in a pH 4.5 or 6 solution caused < 5% change in fluorescence intensity.

**Figure 8.**
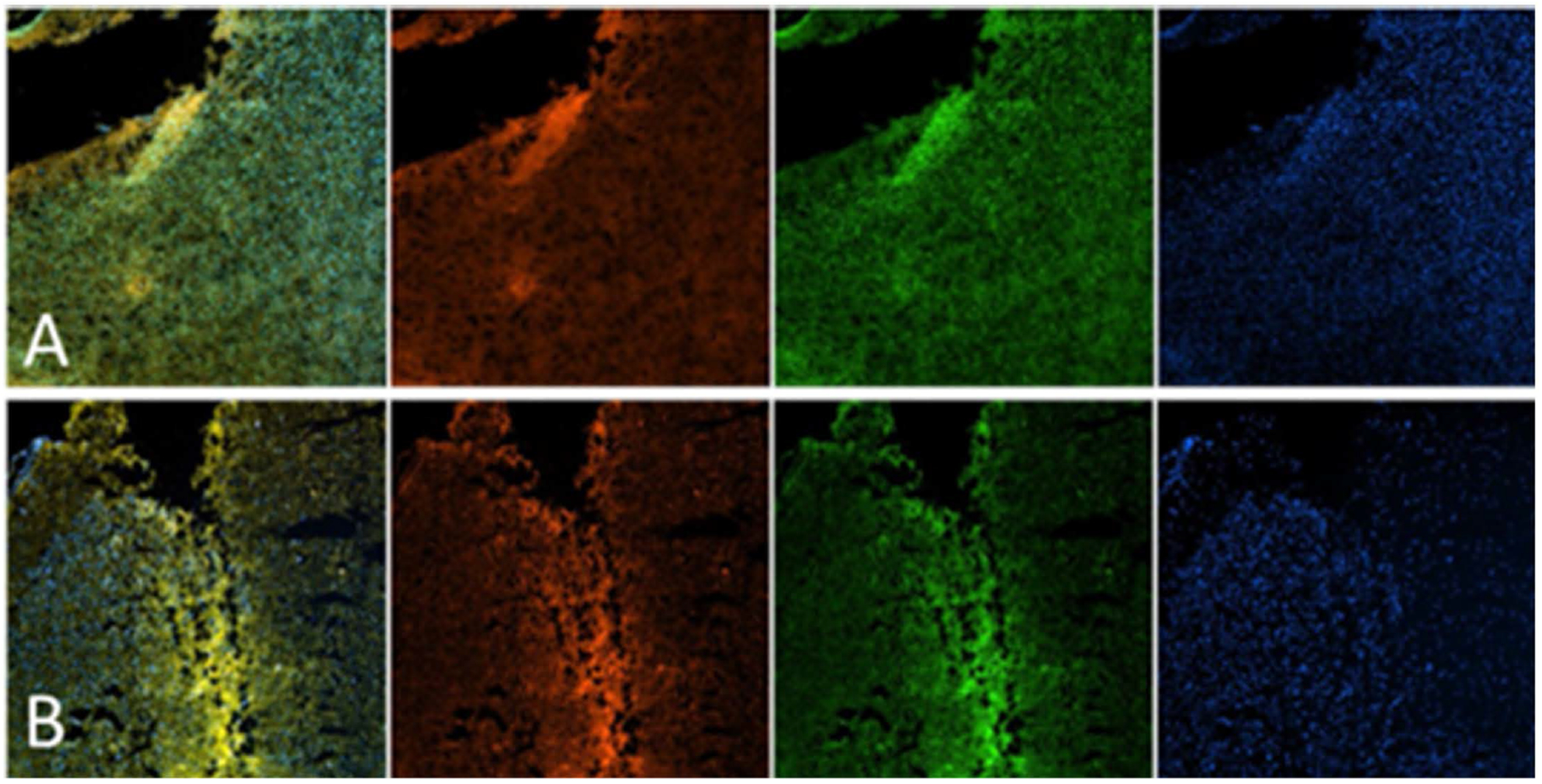
Fluorescent P188 can cross the blood-brain barrier. Eleven-week-old male CD1 male mice underwent (A) sham injury or (B) mild TBI and were treated with either vehicle (not shown) or 15 μg/mouse of P188 + Rh110 (red) intravenously 1 hour after injury. At 24 hours, animals were sacrificed, and brain sections were stained with Nissl stain (green) to identify neurons and DAPI (blue) to identify nuclei. All images were acquired using a 20X microscope objective.

Finally, we recently conducted *in vivo* experiments using mice, where P188 + Rh110 (15 μg/mouse) was injected via the tail vein one hour after inducing a moderate TBI injury using a Benchmark Stereotaxic Impactor (Leica Microsystems, Wetzler, Germany). Sham was treated with a vehicle. At 24 hours, brain tissues were harvested, and frozen sections were then stained for neurons and nuclei (Figure 8). Our findings indicate that P188 was able to cross the BBB in both sham and TBI mice, demonstrating widespread distribution throughout the brain. Because the mild TBI did not appear to produce significant differences between the sham and TBI groups, it may be necessary to consider repetitive or more severe TBI for clearer distinctions that could lead to laying a foundation for the development of potential targeted therapeutics. However, the analysis of the damaged brain slice revealed an increased accumulation of P188. This study represents one of the limited reports available in the literature confirming that P188 can cross the BBB in mice.

## 5. CONCLUSION

FDA-approved triblock polymers P188 have been proven to reseal the leaky cell membrane, repair cellular damage in response to traumatic injury, and restore the cellular function of brain endothelial cells [1, 12]. While the reparative mechanisms for P188 continue to be better elucidated, the cellular uptake of P188 is now demonstrated in this paper, along with the identification of likely transport mechanisms. Surprisingly, P188 transport is not through simple diffusion but relies on the traditionally established endocytic pathways, including the clathrin-mediated internalization, and requires an intact cytoskeletal network. Internalized P188 + Rh110 complex was found to be retained in the injured cells and hypothesized to colocalize with lysosomes, indicating the reparative mechanisms may involve the lysosome-mediated cell membrane resealing [44]. Remarkably, the P188 + Rh110 complex was observed to cross the BBB in a mouse model. To the authors’ knowledge, this is the first time that fluorescent P188 + Rh110 has been demonstrated to enter the brain tissue through the BBB. Much more *in vivo* work is required to establish the efficacy of P188 to repair traumatic brain injuries to the brain. Finally, the complexity of the P188 transport dynamics led us to the development of a computer vision pipeline that significantly enhanced image analysis. Formulation of working hypotheses is now feasible with the implementation of the AI-assisted pipeline to segment and track individual cells over time series images or single snapshots. A combination of experimental and computational approaches may enable us to optimally modulate the cellular uptake of FDA-approved P188 and test novel treatment modalities or perhaps preventive measures against traumatic tissue injuries. This easily adaptable machine learning pipeline is indeed expediting the discovery and optimal therapeutic application of repurposing FDA-approved drugs.
